# Peritoneal Sarcomatosis Secondary to a Gastrointestinal Stromal Tumor Treated With a Multimodal Approach: A Case Report

**DOI:** 10.7759/cureus.101456

**Published:** 2026-01-13

**Authors:** Arleyson Daniel Perez Zambrano, Mauricio Garcia Mora, Stefany Rios Acuña

**Affiliations:** 1 Surgical Oncology, Nueva Granada Military University, Bogotá, COL; 2 Surgical Oncology, National Cancer Institute, Bogotá, COL; 3 General Surgery, University of La Sabana, Bogotá, COL; 4 General Surgery, National Cancer Institute, Bogotá, COL

**Keywords:** cytoreduction, gastrointestinal stromal tumor, gist, hipec, imatinib, multimodal treatment, peritoneal sarcomatosis

## Abstract

We report the case of a 49-year-old woman diagnosed with peritoneal sarcomatosis secondary to an epithelioid gastrointestinal stromal tumor (GIST) (Ki-67 3%, CD117 and DOG1 positive), initially managed with empirical carboplatin-paclitaxel, which was discontinued after histological confirmation. Treatment with imatinib 400 mg/day was initiated, achieving sustained metabolic response and clinical stability. After 12 months of targeted therapy, the patient underwent cytoreductive surgery (CRS) with hyperthermic intraperitoneal chemotherapy (HIPEC) using mitomycin C. Intraoperative findings revealed extensive peritoneal disease with distal jejunal involvement, achieving CC-1 cytoreduction. The postoperative course was uneventful, and pathology confirmed a metastatic GIST with low mitotic activity. At one-year follow-up, the patient remains clinically stable, with preserved functional status and no evidence of radiological progression. This case illustrates the potential role of a multimodal strategy combining targeted therapy, CRS, and HIPEC in selected patients with peritoneal involvement from GISTs.

## Introduction

A gastrointestinal stromal tumor (GIST) is the most common sarcoma of the digestive tract, arising from the interstitial cells of Cajal and characterized by activating mutations in the KIT or PDGFRA genes, present in more than 85% of cases [1]. It most frequently occurs in the stomach (55-60%) and the small intestine (30%). Although most cases are diagnosed as localized disease, 10-15% present with metastatic involvement at the time of diagnosis, with the peritoneum and liver being the most common sites of dissemination [1]. The epithelioid variant of GISTs, which is less common than the spindle-cell subtype, displays a particularly rare pattern of peritonealization, increasing the clinical and scientific relevance of this presentation and underscoring the need for systematic documentation.

Peritoneal sarcomatosis secondary to GISTs is an uncommon presentation but carries a poor prognosis, historically associated with survival rates below 20 months in the pre-imatinib era. The development of tyrosine kinase inhibitors (TKIs), particularly imatinib, sunitinib, regorafenib, and ripretinib, has transformed the disease course, extending overall survival beyond five years and converting a previously lethal condition into a potentially controllable scenario [1].

However, in patients achieving partial or stable response to TKIs, the role of complete cytoreductive surgery (CC-0/CC-1) combined with hyperthermic intraperitoneal chemotherapy (HIPEC) has become increasingly relevant as an adjunctive therapeutic strategy. Recent evidence supports the notion that, in specialized centers and with appropriate case selection, this multimodal approach may improve survival and reduce the risk of secondary resistance. International consensus statements, such as the 2020 Chicago Consensus and the 2024 Ibero-American Consensus on Peritoneal Sarcomatosis, recognize the value of integrating targeted systemic therapy with peritoneal oncologic surgery, highlighting the importance of multidisciplinary teams and decision-making guided by tumor biology and treatment response [2,3].

## Case presentation

A 49-year-old woman presented with progressive pelvic pain of several months' duration, associated with abdominal distension and increased girth. She reported no significant weight loss, gastrointestinal bleeding, or constitutional symptoms. On admission, her BMI was 21.9 kg/m², with no relevant chronic comorbidities and a remote history of basal cell carcinoma resected in 2016.

**Figure 1:**
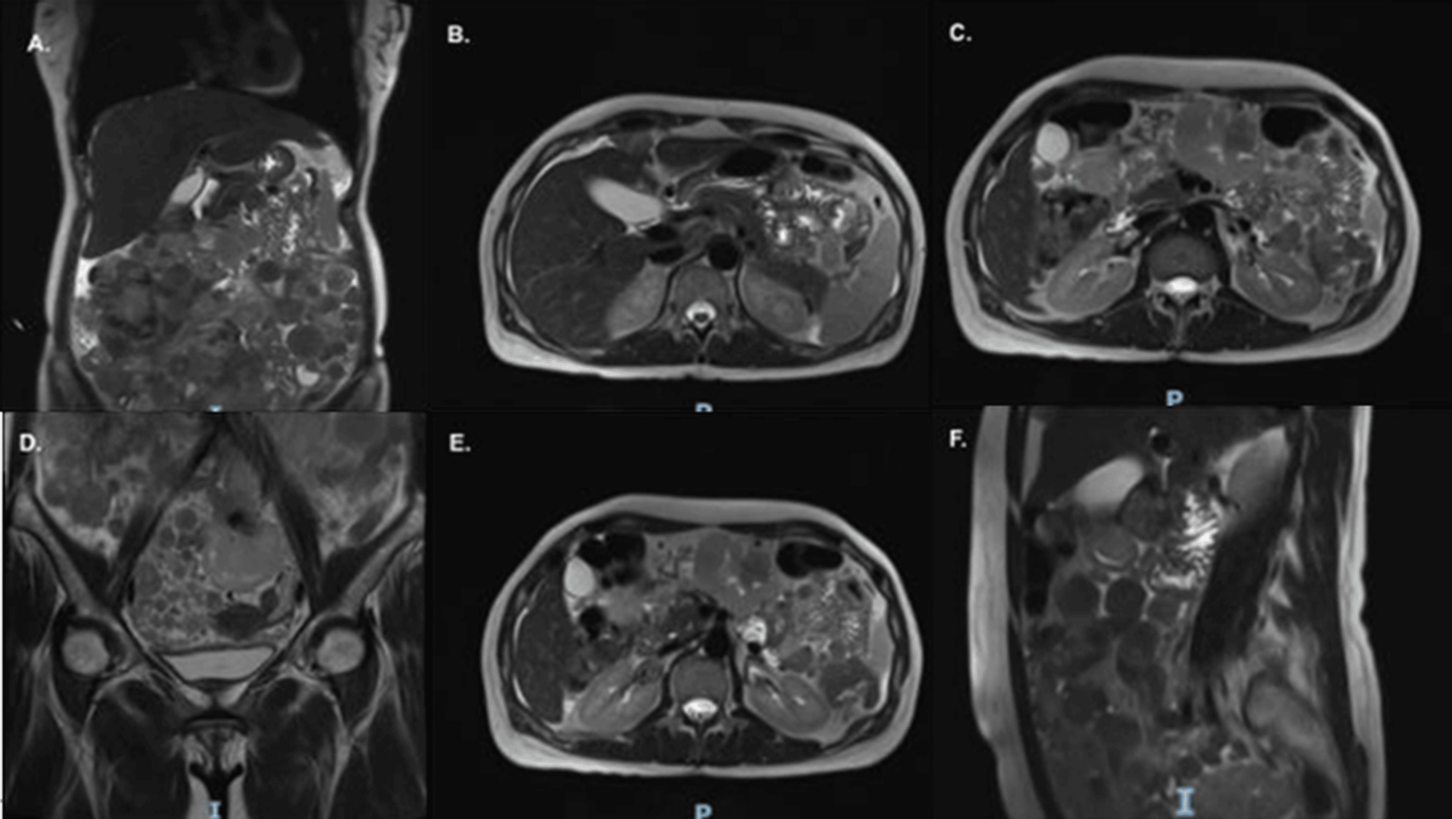
Pre-treatment abdominopelvic MRI (T2-weighted sequences) demonstrating extensive peritoneal sarcomatosis. (A) Coronal view showing diffuse hyperintense peritoneal implants. (B, C, F) Axial and sagittal views confirming widespread peritoneal disease with necrotic components. (D) Coronal T2-weighted image showing pelvic extension of hyperintense peritoneal implants with mild bowel compression. (E) Axial T2-weighted image demonstrating multiple inframesocolic hyperintense peritoneal masses with central necrotic areas.

Baseline laboratory tests showed adequate hematologic reserve and preserved organ function. Imaging studies, including abdominal and pelvic CT and chest CT, documented peritoneal sarcomatosis with calcified solid masses and left adnexal involvement, without evidence of a primary gastrointestinal lesion. Abdominopelvic MRI with contrast (October 2022) showed multiple predominantly solid peritoneal and mesenteric lesions, some with areas of necrosis, the largest measuring 60 mm at the root of the mesentery anterior to the superior mesenteric vessels. Additionally, lesions were observed over hepatic segment IV and in the hepatic hilum, without intestinal obstruction or metastatic liver lesions. The pelvis revealed numerous solid nodules with restricted diffusion, up to 78 mm in size, consistent with extensive peritoneal sarcomatosis of mesenchymal origin (Figure 1). PET-CT was not performed during the initial evaluation. Upper digestive endoscopy reported erosive antral gastritis without suspicious masses or ulcers, and colonoscopy ruled out significant colonic pathology, supporting the impression of a peritonealized GIST without a clear primary origin detectable in conventional studies.

The initial histological study of an incisional peritoneal biopsy revealed a GIST with an epithelioid pattern (Ki-67 3%), positive for CD117 and DOG1, and negative for keratins, S100, and calretinin. This is a GIST of unidentified primary origin, with peritoneal presentation.

The predominant mutational study (KIT and PDGFRA) was not available in the patient’s initial clinical documentation. In this context, and given that molecular characterization is not always routinely integrated in resource-limited institutions, imatinib was initiated based on the characteristic immunohistochemical profile (CD117/DOG1 positive) and clinical presentation. The initiation of imatinib without prior mutational profiling, while supported by strong immunohistochemical evidence, represents a deviation from ideal practice and constitutes a limitation of this case, as approximately 10-15% of GISTs harbor PDGFRA mutations or are wild-type, which may influence sensitivity to TKIs. No mitotic figures were identified in 25 high-power fields. Based on these findings, the empirical cytotoxic chemotherapy (carboplatin-paclitaxel) administered at another institution was discontinued, and imatinib 400 mg/day was started as first-line treatment.

The patient showed adequate tolerance and a documented partial response in serial imaging controls, with a decrease in the volume of peritoneal implants and clinical stability.

**Figure 2:**
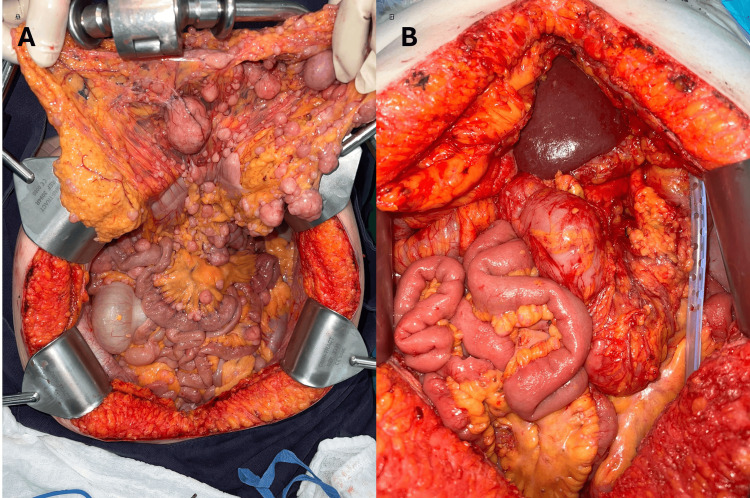
Intraoperative findings. (A) Extensive pre-cytoreduction peritoneal tumor burden involving the mesentery and visceral surfaces. (B) Post-cytoreduction surgical field showing complete macroscopic tumor removal (CC-1), prior to HIPEC. HIPEC: Hyperthermic intraperitoneal chemotherapy

During oncological follow-up, in June 2024, she was considered a candidate for cytoreductive surgery (CRS) due to sustained metabolic control and absence of resistance mutations. Exploratory laparotomy revealed extensive peritoneal carcinomatosis (PCI 21) with multiple implants in the diaphragm, pelvis, mesentery, and distal small intestine. Complete cytoreduction (CC-1) was performed with radical omentectomy, supramesocolic and inframesocolic peritonectomy, pelviperitonectomy, hysterectomy with bilateral salpingo-oophorectomy, segmental resection of the distal jejunum (40 cm), and mechanical side-to-side anastomosis (Figure 2). Subsequently, HIPEC with mitomycin C (15 mg/m² at 43 °C for 60 minutes) was administered via an open technique, without intraoperative complications or bile leaks.

The postoperative period was uneventful; the patient resumed progressive oral tolerance and was discharged in good general condition. The histopathological study of the surgical specimens confirmed metastatic involvement by a spindle cell GIST in the mesosalpinx, diaphragmatic dome, distal jejunum, omentum, colonic, pelvic, and mesenteric implants; all samples were positive for CD117 and DOG1, with a mitotic index of 0-1 figures in 5 mm², and negative surgical margins.

In subsequent imaging studies (December 2024 and June 2025), abdominal and pelvic MRIs revealed no tumor progression, resolution of free fluid, and stability of an extraperitoneal scar nodule in the left iliac fossa, interpreted as a post-surgical change. No new implants or metastatic liver lesions were reported. One year after the intervention, the patient remains on continuous treatment with imatinib 400 mg/day, with ECOG 1, Karnofsky 90%, without adverse events or radiological relapse, maintaining a sustained partial response and adequate quality of life.

## Discussion

Peritoneal sarcomatosis secondary to a GIST presents a complex clinical challenge due to its rarity, molecular heterogeneity, and unpredictable biological behavior. In the pre-TKI era, the prognosis for patients with metastatic disease was grim, with survival rarely exceeding 20 months [4]. The introduction of imatinib mesylate, the first TKI approved for GISTs, revolutionized management by specifically blocking the KIT or PDGFRA receptor signaling pathway, responsible for more than 85% of cases [1].

Our case involves the less common epithelioid variant of GISTs presenting as primary peritoneal sarcomatosis with an occult primary tumor, highlighting both the diagnostic challenge and the critical role of immunohistochemistry in guiding initial systemic therapy.

Advances in targeted therapy have transformed metastatic GISTs into a manageable chronic disease, with median overall survival now exceeding five years in patients treated sequentially with imatinib, sunitinib, regorafenib, and ripretinib [5]. Recent studies, such as that by Tsai et al., report a four-year overall survival rate of 79.5% and a progression-free survival rate of 50.5% in real-world cohorts of patients with metastatic GISTs treated with TKIs [6]. These results highlight the importance of molecular stratification, treatment adherence, and multidisciplinary follow-up.

However, a subgroup of patients develops peritoneal dissemination as the sole metastatic site. In such cases, the combination of systemic therapy with CRS and HIPEC has emerged as a potentially effective option for achieving prolonged control in specialized centers. Several series, although retrospective, show that when complete cytoreduction (CC-0/CC-1) is achieved, survival can be doubled compared to medical therapy alone [7].

The Ibero-American Consensus for the Management of Peritoneal Sarcomatosis (2024) proposes that CRS-HIPEC should be considered in patients with peritoneal GISTs when there is a good systemic response, a PCI score ≤ 20, and the possibility of complete resection. In its review of 33 studies, the complete cytoreduction rate reached 87%, and the five-year survival rate was 35-65%, with acceptable morbidity (<20%) [2]. Similarly, the Chicago Consensus 2020 recommended that in tumors of mesenchymal origin, such as GISTs, HIPEC with mitomycin C or cisplatin can be used as an adjuvant strategy after radical CRS in the absence of extra-abdominal disease [3].

The accumulated experience in peritoneal sarcomatosis supports this approach. The meta-analysis by Helm et al., which included 320 patients with different sarcoma subtypes treated with CRS-HIPEC, showed a five-year overall survival rate of 35% and a major morbidity rate of 17%, concluding that the benefit depends on the degree of cytoreduction and the initial tumor volume [8]. In the specific context of GISTs, Medina et al. described “peritoneal gistosis” as an entity where complete resection and intraperitoneal perfusion with mitomycin C contribute to locoregional control [9].

Although the patient’s peritoneal cancer index (PCI) of 21 slightly exceeded the threshold suggested by some consensus guidelines, the excellent response to imatinib and the feasibility of achieving CC-1 cytoreduction supported proceeding with CRS-HIPEC. This underscores that the PCI should be interpreted within a multifactorial decision-making model that also incorporates treatment response, disease biology, and surgical resectability.

The case presented is consistent with this contemporary evidence: after 12 months of treatment with imatinib 400 mg/day and a partial radiographic response, CRS-HIPEC was performed with mitomycin C, achieving CC-1, without complications and with prolonged clinical stability. These results are consistent with those described by Sommariva et al. and Rubió-Casadevall et al., who reported superior survival rates when surgery is performed in the context of systemic control and with negative margins [10,11].

Likewise, recent reviews emphasize that salvage or CRS in metastatic GISTs should be reserved for patients with a good radiological or biological response to TKI, low surgical risk and possibility of complete resection, since surgery in progressive disease is associated with worse outcomes [11,12].

From a biological perspective, reducing tumor burden through CRS may decrease the absolute number of tumor cells at risk of developing secondary resistance mutations, particularly in KIT exons 13 and 17 [13], thereby potentially prolonging the effectiveness of adjuvant imatinib. This rationale is consistent with the tumor volume-dependent outcomes reported in the meta-analysis by Helm et al. [8]. Furthermore, this concept is supported by the observation that post-imatinib progression often arises from residual peritoneal implants, which may be eliminated through CRS-HIPEC.

Finally, appropriate patient selection is essential. The European Society for Medical Oncology (ESMO) and the GEIS 2023 Group recommend that the surgical approach for metastatic GISTs be decided by multidisciplinary committees at referral centers, considering the mutation, TKI response, PCI score, and functional status [1]. In this context, the patient described (ECOG 1, PCI score 21, sustained partial response) represented an optimal candidate for sequential multimodal treatment.

Current evidence supports the integration of targeted therapy, surgical cytoreduction, and HIPEC, which can offer prolonged survival and durable cancer control in selected patients with peritoneal GISTs. Although randomized studies are scarce, the results of multiple series and international consensus statements agree that CRS-HIPEC, performed in experienced centers, is a valid and safe alternative within an individualized, multimodal approach.

The therapeutic sequence illustrated in this case, neoadjuvant TKI therapy to achieve disease control and assess biological behavior, followed by consolidative CRS-HIPEC and continuation of adjuvant TKI, reflects the emerging paradigm for selected patients with advanced peritoneal involvement from GISTs.

## Conclusions

The present case illustrates that a multimodal strategy integrating imatinib-based targeted therapy, CRS, and HIPEC can achieve sustained disease control and preserve quality of life in carefully selected patients with peritoneal sarcomatosis secondary to GISTs. Rigorous patient selection, multidisciplinary decision-making, and management in highly specialized centers are essential to optimize oncologic outcomes and minimize morbidity. This experience supports the role of CRS-HIPEC as a valid therapeutic option within a personalized multimodal approach for GISTs confined to the peritoneum.
